# Synthesis of Fe_3_C@C from Pyrolysis of Fe_3_O_4_-Lignin Clusters and Its Application for Quick and Sensitive Detection of PrP^Sc^ through a Sandwich SPR Detection Assay

**DOI:** 10.3390/ijms20030741

**Published:** 2019-02-10

**Authors:** Chenglong Yuan, Zhichao Lou, Weikai Wang, Lintian Yang, Yanjun Li

**Affiliations:** College of Materials Science and Engineering, Nanjing Forestry University, Nanjing 210039, China; m18331276712@163.com (C.Y.); zc-lou2015@njfu.edu.cn (Z.L.); 15261863591@163.com (W.W.); ylt921028@163.com (L.Y.)

**Keywords:** surface plasmon resonance, prion protein, aptamer, sensitive, Fe3C, core/shell

## Abstract

The prion protein (PrP^Sc^) has drawn widespread attention due to its pathological potential to cause prion diseases. Herein, we successfully synthesized Fe_3_C@C by carbonizing Fe_3_O_4_-lignin clusters, which were prepared through a facile hydrogen bonding interaction between ≡Fe-OH and hydroxyl groups of lignin. Our in-depth investigation confirmed that the composites were Fe_3_C@C core/shell particles. We constructed a novel sandwich surface plasmon resonance (SPR) detection assay for sensitive PrP^Sc^ detection, utilizing bare gold surface and aptamer-modified Fe_3_C@C (Fe_3_C@C-aptamer). Due to the highly specific affinity of Fe_3_C@C-aptamer towards PrP^Sc^, the sandwich type SPR sensor exhibited excellent analytical performance towards the discrimination and quantitation of PrP^Sc^. A good linear relationship was obtained between the SPR responses and the logarithm of PrP^Sc^ concentrations over a range of 0.1–200 ng/mL. The detection sensitivity for PrP^Sc^ was improved by ~10 fold compared with the SPR direct detection format. The required detection time was only 20 min. The specificity of the present biosensor was also confirmed by PrP^C^ and other reagents as controls. This proposed approach could also be used to isolate and detect other highly pathogenic biomolecules with similar structural characteristics by altering the corresponding aptamer in the Fe_3_C@C conjugates.

## 1. Introduction

The prion protein has drawn great attention due to its pathological potential to cause prion diseases, which are recognized as highly contagious and scarcely incurable [1]. The prion protein has two isoforms, PrP^C^ and PrP^Sc^, which are with the same amino acid sequence but with different conformations. PrP^C^ is proved to be a kind of host-encoded normal protein [2,3] which will transfer to the pathological conformer (PrP^Sc^) in certain conditions, and PrP^Sc^ will form the extended β-sheet rich fibrillary PrP aggregates [4]. On the other side, PrP^Sc^ can also introduce the transformation of normal PrP^C^, thus PrP^Sc^ is supposed to be a marker for transmissible spongiform encephalopathy infections and to be a causative agent [5,6]. Based on this, discriminate and quantitative detection of trace PrP^Sc^ is in urgent need for prion disease diagnosis and the monitoring of disease treatment.

For decades, numerous traditional methods have been introduced for the detection of PrP^Sc^, such as protein misfolding cyclic amplification [7], ELISA [8], micromechanical resonator arrays [9], fluorescence correlation spectroscopy [10], fluorescence in situ hybridization (FISH) [11], and immune-quantitative real-time PCR assays [12]. These methods have been proven to be with good sensitivity and some of them are commonly used in practical tests. However, these methods normally require practiced experimental skills and expensive instruments, and are time- and labor-intensive, making them difficult to be used for the high-throughput detection or routine testing. Compared with these methods, surface plasmon resonance (SPR) biosensors are supposed to be commercialized approaches with higher precisions and sensitivities, which have been used in the field of theranostics (therapeutics and diagnostics), pharmaceutics, food safety, environmental monitoring and homeland security [13,14]. It is important for the fabrication of a novel SPR-based detection assay for the detection of PrP^Sc^ with ultra-sensitivity and high-specificity.

Nowadays, nanomaterials have drawn a great deal of attention because of their potential applications for improving the detection capacities of SPR, since many scientific reports suggest that a lower limit of detection and a wider detection range can be obtained when integrating nanomaterials into a miniaturized SPR system [15,16]. Among these nanomaterials, magnetic nanoparticles (MNPs) are used for SPR signal amplification, based on their special advantages. For example, their high refractive index and high molecular weight, which effectively increase the SPR signals in the sandwich SPR system [17,18]. Our group has developed a novel sandwich SPR detection assay for ultrasensitive detection of PrP^Sc^ using PrP^Sc^ conjugating magnetic nanoparticle (Fe_3_O_4_) clusters as signal amplification reagents [19]. However, in normal usage Fe_3_O_4_ is easily oxidized into Fe_2_O_3_, which changes its magnetic and surface chemical properties, and thus reduces its efficiency of target molecule separation and affects the modification stability of the distinguishing probes on its surface. One approach to solve this problem is to load other materials to fabricate multiphase composite magnetic materials. Due to the advantages of abundant resources, good electric properties, facile manipulation and relatively excellent chemical and thermal stability, carbon materials are one of the most promising candidates to surround MNPs.

Herein, we firstly synthesized Fe_3_C@C core-shell MNPs by carbonizing Fe_3_O_4_-lignin hybrids which were prepared through facile hydrogen bonding interactions. From the environmental point of view, the usage of lignin as the carbon resource is of positive significance, since lignin is a by-product of the pulping and papermaking industry, with world production of around 30 million tons per year. A novel sandwich SPR biosensor was then constructed for sensitive PrP^Sc^ detection using aptamer-modified Fe_3_C@C (Fe_3_C@C-aptamer) as a recognition and amplification reagent for enhancement of the SPR signal. The sandwich type SPR sensor here exhibited excellent analytical performance towards the quantification and quantitation of PrP^Sc^, with high sensitivity and good selectivity. Atomic force microscope (AFM) was used to investigate the surface morphology of the SPR substrate after detection. This proposed approach can also be used to detect other analytes by altering the corresponding aptamer in the Fe_3_C@C conjugates.

## 2. Results and Discussion

**Figure 1:**
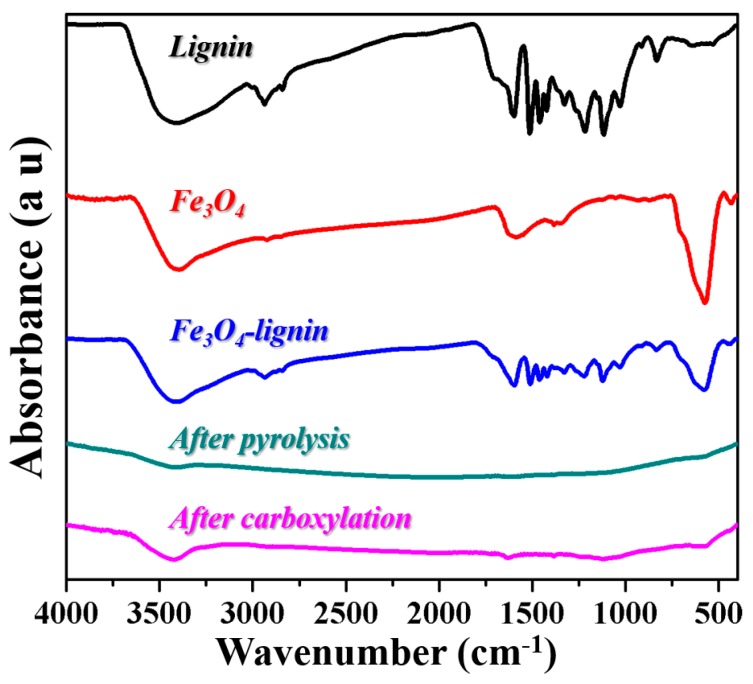
FTIR curves of lignin, Fe_3_O_4_, Fe_3_O_4_-lignin, and the samples after the pyrolysis and carboxylation processes.

The functional groups of lignin, Fe_3_O_4_, synthesized Fe_3_O_4_-lignin, and the samples after pyrolysis and subsequent carboxylation processes were investigated by FTIR analysis. The corresponding FTIR spectra are shown in Figure 1. As compared with lignin and Fe_3_O_4_, the bands observed between 400 cm^−1^ and 600 cm^−1^ for Fe_3_O_4_-lignin were associated with the stretching and torsional vibration modes of the magnetite. The vibrational bonds of the aromatic ring were observed at 1592 cm^−1^ and 1508 cm^−1^, and two other peaks appeared at 1461 cm^−1^ and 1417 cm^−1^, attributing to the methoxyl groups in lignin structure. Since the product was obtained by magnetic separation, these results here indicated that the natural lignin was evidently modified on the Fe_3_O_4_ nanoparticles. After the pyrolysis process, we saw from Figure 1 that the absorption bands mentioned above for the modified lignin could be hardly detected. Additionally, the absorption peak representing Fe_3_O_4_ also disappeared after the pyrolysis process. However, after the subsequent carboxylation process, we observed two additional peaks at 1629.53 cm^−1^ and 1382.69 cm^−1^ which were supposed to be the representing absorption peaks for the –COOH group, which indicated a successful carboxyl functionalization.

**Figure 2:**
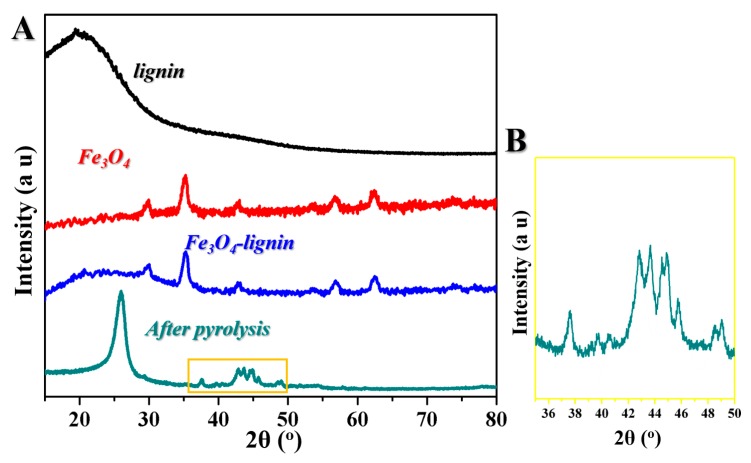
(**A**) XRD curves of lignin, Fe_3_O_4_, Fe_3_O_4_-lignin, and the samples after the pyrolysis process. (**B**) Magnified XRD curve of the samples after the pyrolysis process.

**Figure 3:**
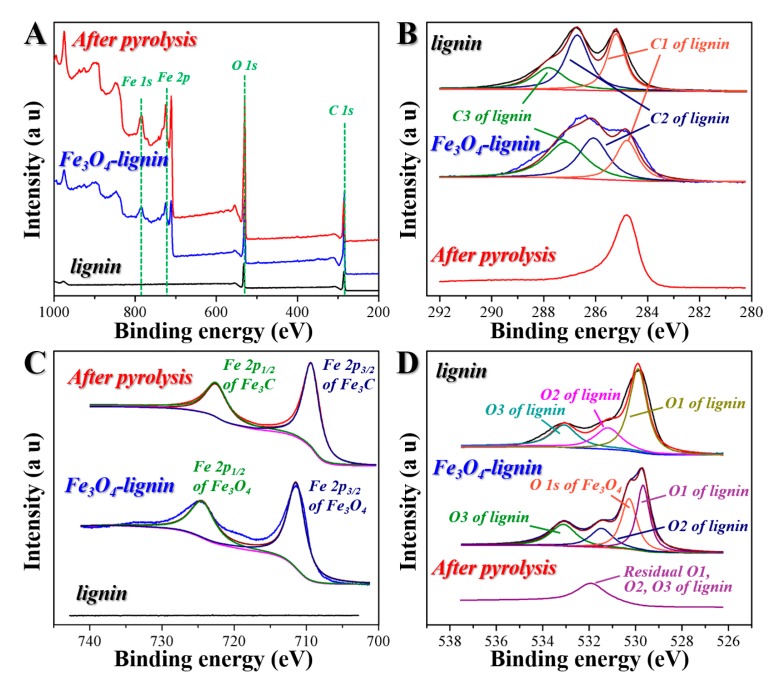
(**A**) X-ray photoelectron spectroscopy (XPS) spectra of lignin, Fe_3_O_4_-lignin and the sample after pyrolysis process. (**B–D**) The corresponding high-resolution XPS spectra in C 1s, Fe 2p and O 1s, respectively.

The crystalline structures of the samples were studied by wide-angle XRD, as shown in Figure 2A. We noticed that both the lignin and Fe_3_O_4_-lignin displayed primary diffraction peaks at around 22.5º, which could be assigned to lignin. Fe_3_O_4_-lignin showed the additional diffraction peaks at 2θ = 30.0º, 35.3º, 43.0º, 53.4º, 56.9º and 62.5º, corresponding to the (220), (311), (400), (422), (511), and (440) planes of Fe_3_O_4_ in a cubic phase, respectively [20]. As shown in Figure 3A and the magnified curve in Figure 2B, after the pyrolysis process, the observed peaks at 2θ = 37.6º, 39.8º, 40.6º, 42.8º, 43.7º, 44.5º, 45.0º, 45.8º, 48.6º, 49.1º and 51.8º corresponded to the atomic plane-reflections of (210), (002), (201), (211), (102), (220), (031), (131), (221) and (122), respectively, which indicated the conversion from Fe_3_O_4_-lignin to Fe_3_C [21]. Furthermore, a sharp peak at 26.1º was observed, corresponding to the graphite-like structure attributed to the carbonization of lignin at 1000 °C.

The XPS measurements were introduced to further confirm the different phases in the samples, as shown in Figure 3. It was obvious that lignin contained mainly C and O, while Fe_3_O_4_-lignin and the pyrolysized sample contained C, O and Fe (Figure 3A). To determine the oxidation states of the elements, high-resolution XPS spectra of C 1s, Fe 2p and O 1s are highlighted in Figure 3B–D. As shown in Figure 3D, the XPS spectra for O of lignin and Fe_3_O_4_-lignin were both broad because of the co-presence of O1 (-C=O), O2 (carbonyl oxygen atoms in esters, amides and anhydrides), and O3 (ether oxygen atoms in esters and anhydrides) from the lignin phase. Additionally, for Fe_3_O_4_-lignin, the XPS spectra for O contained the O1s at ~530.5 eV for Fe_3_O_4_. After the pyrolysis process, the O lineshape became sharper, which indicated the pyrolysis of the modified lignin, and the representing peak for Fe_3_O_4_ disappeared, which indicated the phase conversion from Fe_3_O_4_ to Fe_3_C. As shown in Figure 3C, the high-resolution XPS Fe 2p spectra for Fe_3_O_4_-lignin showed double satellite signals at ~711.0 eV and ~725.0 eV, corresponding to the characteristic doublet from Fe 2p_3/2_ and Fe 2p_1/2_ core-level electrons in the Fe_3_O_4_ phase. After the pyrolysis process, the peaks of Fe 2p shifted from high binding energies to low ones at ~722.7 eV and ~709.1 eV, which were the characteristic doublet for Fe_3_C [22]. This was in accordance with the results of the XRD. The peak at around 285.0 eV in Figure 3B was attributed to C 1s, and the observed variation was similar to the one for O1s. In detail, for lignin and Fe_3_O_4_-lignin, the corresponding spectra were broad because of the presence of C-C, C-H, C-O and C=O groups, which were in accordance with the FTIR results. After the pyrolysis process, the C lineshape became sharper and was attributed to the carbonization of lignin along with the formation of Fe_3_C.

**Figure 4:**
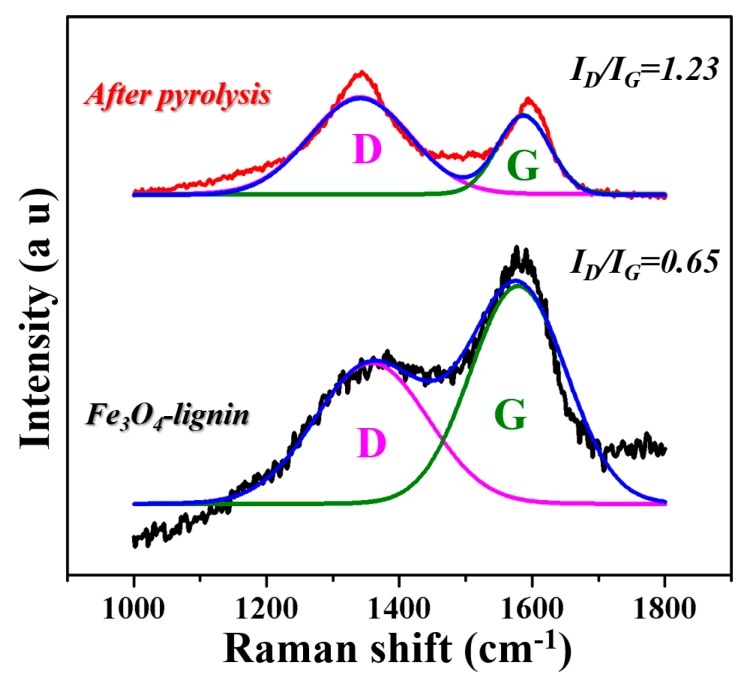
Raman spectra of Fe_3_O_4_-lignin and the sample after pyrolysis.

The graphitization degrees of the samples were investigated by Raman spectroscopy because of its sensitivity to sp2 carbon structures on the nanometer scale. The typical Raman spectra of Fe_3_O_4_-lignin before and after the pyrolysis processes are shown in Figure 4. Both samples exhibited two bands near 1340 cm^−^1 and 1590 cm^−^1, referring to the vibration of sp3 atoms (D-band) and the in-plane vibration of sp2 atoms (G-band), respectively. The peak area ratio of *I_D_*/*I_G_* has been extensively used as an important parameter to study the crystalline or graphite-like carbon structures and a higher *I_D_/I_G_* value means a higher graphitization degree to the amorphous state of carbon. Here, an increased peak area ratio of *I_D_/I_G_* value from 0.65 to 1.23 was obtained after the pyrolysis process, implying the conversion of lignin into a more ordered, graphite-like carbon.

**Figure 5:**
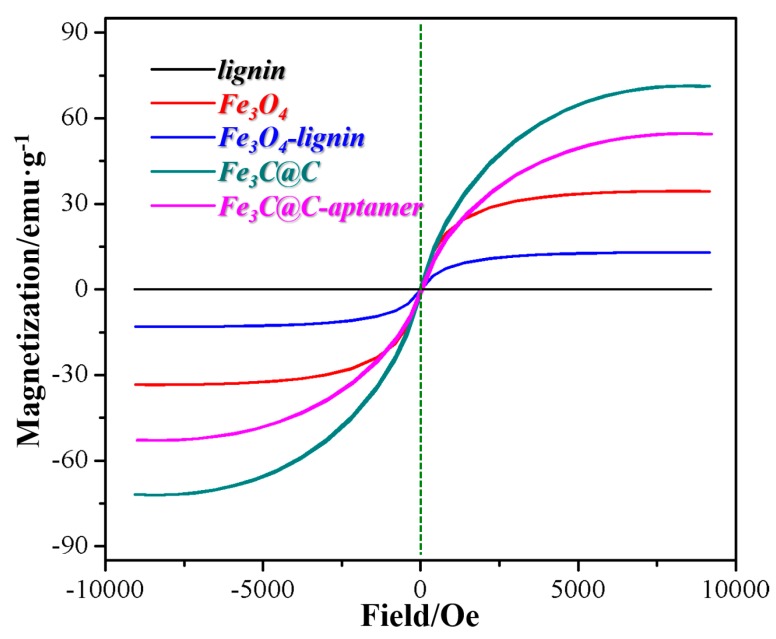
VSM curves of lignin, Fe_3_O_4_, Fe_3_O_4_-lignin, Fe_3_C@C and Fe_3_C@C-aptamer.

The magnetic properties of the prepared lignin, Fe_3_O_4_, Fe_3_O_4_-lignin, Fe_3_C@C and Fe_3_C@C-aptamer were measured by VSM, as shown in Figure 5. As can be seen, the typical characteristics of magnetic behavior were observed. The corresponding saturation magnetization values were 0 emu/g, 34.46 emu/g, 13.03 emu/g, 71.20 emu/g and 54.56 emu/g, respectively. The decrease of saturation magnetization value from Fe_3_O_4_ to Fe_3_O_4_-lignin and from Fe_3_C@C to Fe_3_C@C-aptamer was due to the modification of lignin/aptamer surrounding the particles, and the increase of saturation magnetization value from Fe_3_O_4_-lignin to Fe_3_C@C was supposed to be due to the reduction in sample mass caused by the carbonization process, since the modified lignin layers are pyrolyzed into graphite at 1000 °C. The results revealed that this as-synthesized Fe_3_C@C-aptamer exhibited good magnetic resonance, suggesting its potential application of magnetic separation for PrP^Sc^ from the infected liquid samples.

**Figure 6:**
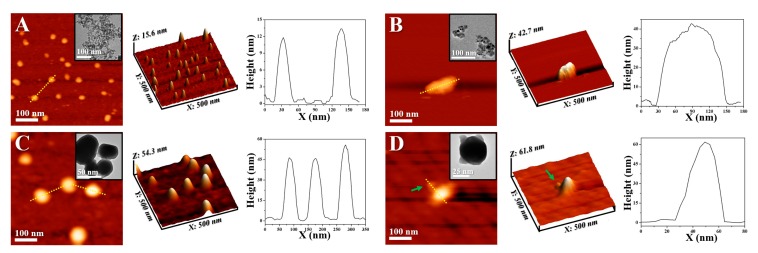
Atomic force microscopy (AFM) images and TEM images (insert) of (**A**) Fe_3_O_4_, (**B**) Fe_3_O_4_-lignin, (**C**) Fe_3_C@C, (**D**) Fe_3_C@C-aptamer, and their corresponding cross-section profiles, respectively.

The morphologies of Fe_3_O_4_, Fe_3_O_4_-lignin, Fe_3_C@C and Fe_3_C@C-aptamer were investigated by AFM and TEM, as shown in Figure 6. Compared with Figure 6A,B, we saw that further chemisorption of natural lignin induced the formation of particles of clusters. This was proposed to be due to the hydrogen bonding interactions between the modified lignin on the surface of Fe_3_O_4_. Figure 6C shows that the Fe_3_C@C synthesized by pyrolysis treatment were spherical under AFM imaging and were of a classical core-shell structure from the different contrast, from the inserted TEM image. As can be seen from the high-resolution AFM image in Figure 6D, we saw a smaller dot surrounding the bright dot, which was in accordance with the TEM result. Based on its height value (1~2 nm) obtained from the cross-section profile, this dot was supposed to be the modified aptamer on the Fe_3_C@C surface. Moreover, the height increase of Fe_3_C@C after the aptamer modification, from ~50 nm to ~60 nm in the field of AFM, also confirmed the successful modification of aptamer molecules.

**Figure 7:**
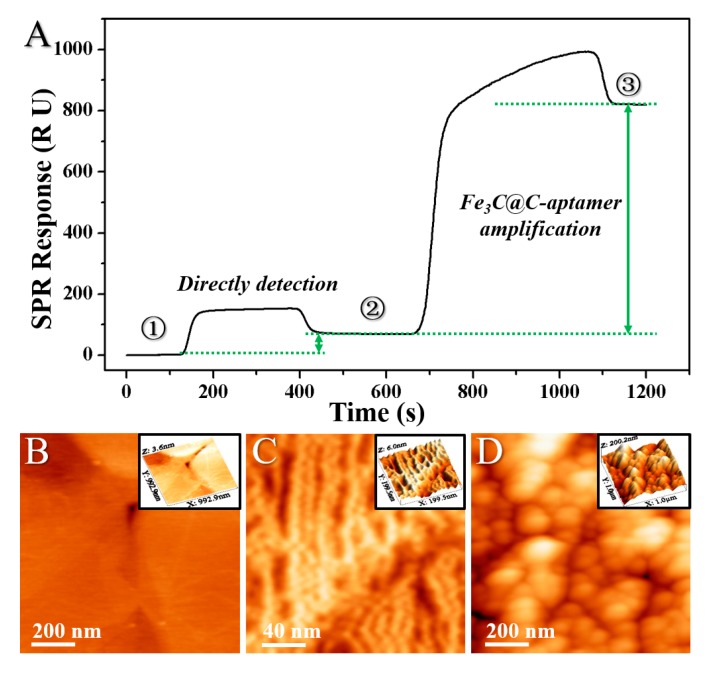
(**A**) The representative real-time SPR sensorgram of the detection of PrP^Sc^ with a concentration of 10 ng/mL before and after the signal amplification by Fe_3_C@C-aptamer conjugate. (**B**–**D**) The AFM images of the sensing films in different stages (labeled as 1, 2, and 3 in Figure 7A) of the detection process and the corresponding 3D AFM images (insert).

**Figure 8:**
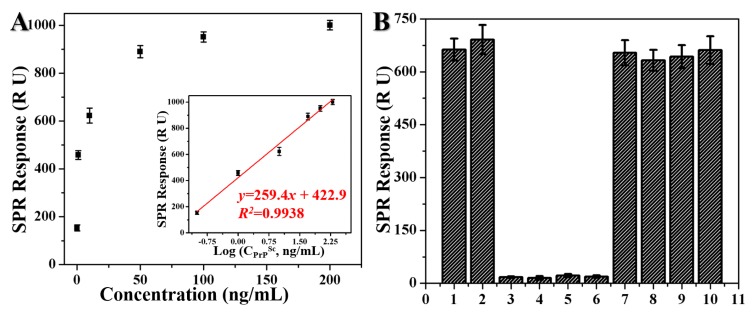
(**A**) SPR response of PrP^Sc^ with concentrations of 0.1 ng/mL, 1 ng/mL, 10 ng/mL, 50 ng/mL, 100 ng/mL and 200 ng/mL, respectively. Insert: Calibration curve of Fe_3_C@C-aptamer involved SPR detection assay. (**B**) Specific analysis of Fe_3_C@C-aptamer involved SPR amplification detection. 1: PrP^Sc^ (10 ng/mL) in PBS buffer; 2: PrP^Sc^ (10 ng/mL) in NBCS; 3–6: MPA, thioPEG, Cys-protein G and PrPC (10 ng/mL each) in PBS buffer, respectively; 7–10: Mixture of PrP^Sc^ (10 ng/mL) and each of the four different reagents (10 ng/mL) in PBS buffer.

The representative real-time SPR sensorgram of the detection of PrP^Sc^ with a concentration of 10 ng/mL before and after the signal amplification by the Fe_3_C@C-aptamer conjugate is shown in Figure 7A, and the AFM images of the sensing films in different stages of the detection process are shown in Figure 8B–D, respectively. As seen in Figure 7A, the Fe_3_C-aptamer involved SPR detection format yields a high enhancement degree of the signal from 68.86 RU (direct detection) to 662.70 RU (Fe_3_C-aptamer amplification) with a time requirement of only 20 min, which indicated high-sensitivity and high efficiency of this method. For an in-depth investigation of the mechanism of the SPR signal changes, high-resolution AFM imaging of the sensing film before and after detection was introduced. As shown in Figure 7B, before the injection of the PrP^Sc^ solution, the bare gold sensing film was clean with obvious gold edges. After injection of the PrP^Sc^ solution into the SPR cuvette, PrP^Sc^ molecules were captured by the gold film via an Au-S bonding interaction between the gold atoms and the exposed disulfide bonds of PrP^Sc^ [23], which induced an SPR response as shown in Figure 7A. From Figure 7C, we observed clear parallel lines composed of PrP^Sc^ following along the gold (111) crystal direction, which confirmed the interactions between the gold atoms and disulfide bonds, and the capture of PrP^Sc^ molecules onto the sensing film. AFM was also used to evaluate the surface morphology of the sensing film after the amplification assay. From the AFM images in Figure 7D, we observed colloids adsorption onto the sensing film, which resulted in further enhancement of the SPR signals as shown in Figure 7A. The modification of the Fe_3_C@C-aptamer conjugates was attributed to the specific interactions between the anti-PrP^Sc^ aptamer (SAF-93) and the captured PrP^Sc^ on the sensing film.

To fully explore the sensitivity of the sandwich detection system, PrP^Sc^ samples with varying concentrations were tested and the results are shown in Figure 8A. From the results, we observed that the SPR response caused by the binding of the Fe_3_C@C-aptamer conjugates increased gradually with increasing PrP^Sc^ concentrations from 0.1–200 ng/mL. A good linear relationship was obtained between SPR responses and the logarithm of the PrP^Sc^ concentrations, as shown inserted in Figure 8A. The regression equation was *y* = 259.4*x* + 422.9 (*R^2^*= 0.9938, *x* is the logarithm of PrP^Sc^ concentration (Log (ng/mL)) and *y* is the SPR signal (RU). The limit of detection (LOD) of this method for PrP^Sc^ was 0.02 ng/mL. The specificity of the amplification detection format was investigated by detection of PrP^Sc^ (10 ng/mL) in both PBS buffer and NBCS, three different reagents (MPA, thioPEG and Cys-protein G, 10 ng/mL) which all have sulfhydryl groups and can assemble on the gold surface, PrP^C^ (10 ng/mL), and the mixture of PrP^Sc^ (10 ng/mL) and each of the four different reagents (10 ng/mL) using the amplification detection format, respectively. The results are shown in Figure 8B, from which it could be observed that PrP^Sc^ had an average response of ~660 R U, which was much greater than that of the other four reagents (~17 R U). In addition, similar SPR responses were obtained for PrP^Sc^ in four mixed samples, which indicated that these four reagents had no effect on the SPR detection response of PrP^Sc^ in a complicated environment. All these results confirmed that the Fe_3_C@C-aptamer conjugate involved SPR approach had good specificity for PrP^Sc^ detection.

## 3. Materials and Methods

### 3.1. Chemicals

Corn stover was obtained in Zhao Dong, Heilongjiang Province, China. β-glucosidase (10–30 units/mg solid, No. G4511), FeCl_3_·6H_2_O (≥99%, No. 31232), FeCl_2_·4H_2_O (≥99%, No. 44939), *N*-(3-Dimethylaminopropyl)-*N*-ethylcarbodiimide hydrochloride (EDC), *N*-hydroxysuccinimide (NHS), HCl (32 wt.%, No. W630574) and NH_3_·H_2_O (25%, No. 32145) were purchased from Sigma-Aldrich (Shanghai, China). The prion protein was purchased from Calbiochem^®^ in Darmstadt, Germany. The transformation from PrP^C^ to PrP^Sc^ was induced by incubation of PrP^C^ in sodium acetate-acetic acid buffer (pH 4.0). PBS (pH 7.2) was purchased from Thermo Scientific in Waltham, MA, USA. Thiol end-functionalized peptide nucleic acid (thiolPNA) and terminal amino functioned anti-PrP^Sc^ aptamer (SAF-93, [24]) with twenty thymine bases as the spacer, which is proved to be of more than 10-fold higher affinity for PrP^Sc^ than for PrP^C^, were synthesized by Shanghai Sangon Biotechnology Co. in China. ThioPEG were purchased from Prochimia Surfaces in Poland. The Cys-protein G (Catalog #: 1002-04) was obtained from Shanghai PrimeGene Bio-Tech Co. in China. Newborn Calf Serum (NBCS) was purchased from ThermoFisher Scientific (Catalog #:1610159). Human serum was purchased from Sigma-Aldrich (St. Louis, MO, USA). All aqueous solutions were prepared with deionized water from a Barnstead Nanopure Diamond Laboratory Water System (18 Mcm Barnstead International, Dubuque, IA, USA).

### 3.2. Preparation of Lignin from Natural Bio-Mass Residues

The 100 g natural corn stover residues were firstly steam-exploded in a 1 L autoclave bomb at 190 °C for 10 min. Then, the pretreated corn solids were washed with distilled water, followed by enzymatic saccharification at a substrate loading of 5% (*w*/*v*) with a cellulase loading of 20 FPU/g glucan supplemented with 3 IU β-glucosidase/g glucan. The experiments were performed in 50 mM citrate buffer (pH 4.8) at 50 °C with gentle shaking at 150 rpm for 48 h. Finally, the remaining solid, which was supposed to be a lignin-rich solid, was washed by distilled water and freeze-dried for further use.

### 3.3. Preparation of Fe_3_O_4_ and Fe_3_O_4_-lignin

Fe_3_O_4_ nanoparticles were firstly prepared via coprecipitation interactions as previously reported [25]. Then, the synthesized lignin (500 mg) was added. The mixture was gently shaken for 5 min, and then reacted under mechanical stirring for 12 h at room temperature. After the reaction, the mixture was separated with a magnet and subjected to three washing cycles (water-methanol), and lignin-modified Fe_3_O_4_ (Fe_3_O_4_-lignin) nanoparticles were obtained by hydrogen bonding interactions between ≡Fe-OH and hydroxyl groups of lignin.

### 3.4. Preparation of Fe_3_C@C Core-Shell Nanoparticles

Under N_2_ flow, the as-prepared Fe_3_O_4_-lignin was placed into a tube furnace. After the air being purged and N_2_ being filled in the furnace for 30 min, the samples were heated to 1000 °C at the heating rate of 5 °C/min, and refluxed for 3 h. After the carbonization process, the samples were cooled down to ambient temperature under N_2_ protection, and the Fe-C core-shell nanoparticles were obtained.

### 3.5. Preparation of Fe_3_C@C-Aptamer Conjugate

The synthesized Fe_3_C@C solution was bath-sonicated for 1 h. Then, 3M NaOH was introduced into the solution and the mixture was bath-sonicated for another 3 h. Afterward, HCl was added to neutralize and the solution was filtered and rinsed, and carboxylic acid functioned Fe_3_C@C (Fe_3_C@C-COOH) was synthesized. Then, EDC (75 mM) and NHS (15 mM) were added into the synthesized Fe_3_C@C-COOH solution and the mixture was bath-sonicated for 30 min. After that, SAF-93 (10 µg, diluted in 200 µL PBS) was introduced and the mixture was bath-sonicated for another 10 min, followed by stirring for 12 h in 4 °C. The final product, Fe_3_C@C-aptamer conjugate, was collected by magnetic separation in PBS buffer.

### 3.6. SPR Detection

The BI-2000 SPR biosensor along with the gold films (50 nm thick) was supplied by Biosensing Inc. (Tempe, AZ, USA)). The standard deviation of the signal for detection of deionized water was about 0.2 RU, and the wavelength of the laser source of the SPR instrument was 630 nm. The gold film was mounted on an SPR prism with the matching oil. Then, PrP^Sc^ samples with different concentrations in PBS buffer were successively injected onto the bare gold sensing film. After the signals were recorded, the Fe_3_C@C-aptamer conjugate was injected into the cuvette to enhance the detection signals. To investigate the specificity and selectivity of the immunoassay, mixture solutions of PrP^Sc^ and six other control reagents were also detected by this method with different molar ratios. All the corresponding SPR signals were obtained in three independently repeated experiments.

### 3.7. Characterization

The TEM images were performed with a JOEL 2100F (200 kV). XRD patterns were obtained on a Bruker D8 Advance powder X-ray diffractometer (Bruker, Karlsruhe, Germany) operated at 40 kV and 40 mA using Cu-Ka radiation (λ = 1.54 Å) with 200 mg of each specimen. The data were collected with a 2θ scanning range of 10°–80°. The IR spectra were recorded by FTIR (Thermo Scientific Nicolet 6700 FTIR spectrometer, Thermo Scientific, Waltham, MA, USA), and each specimen together with KBr was pressed to form a tablet. The X-ray photoelectron spectroscopy (XPS) spectrum was measured in an AXIS UltraDLD (Shimadzu, Kyoto, Japan) using an Al Ka X-ray source and operated at 150 W. The magnetic measurements were carried out using a Lake Shore 7407 VSM provided by East Changing Technologies, Inc (Beijing, China). Each sample was dried in vacuo and weighed 10 mg. The atomic force microscopy (AFM) images of the materials and the sensing films were obtained by an Agilent 5500 Controller combined with a 50 µm by 50 µm Agilent multipurpose AFM scanner. Silicon cantilevers tip with a spring constant of around 0.1 N/m were used for experiments. The images were acquired by using the Agilent magnetic AC (MAC) mode AFM with a magnetically coated cantilever. The obtained AFM images were processed by WSxM software.

## 4. Conclusions

This is the first report on the synthesis of Fe_3_C@C core/shell particles from the Fe_3_O_4_-lignin clusters through a pyrolysis process. We constructed a novel sandwich SPR detection assay for sensitive PrP^Sc^ detection, utilizing bare gold surface and aptamer modified Fe3_C_@C (Fe3_C_@C-aptamer). The sandwich type SPR sensor exhibited excellent analytical performance towards the discrimination and quantitation of PrP^Sc^ due to the highly specific affinity of the Fe_3_C@C-aptamer towards PrP^Sc^. A good linear relationship was obtained between SPR responses and the logarithm of PrP^Sc^ concentrations over a range of 0.1–200 ng/mL. The detection sensitivity for PrP^Sc^ was improved by ~10 fold compared with the SPR direct detection format and the required detection time was only 20 min, indicating the high sensitivity and good efficiency of the detection format. The specificity of the present biosensor was also confirmed by PrP^C^ and other reagents as controls. This proposed approach could also be used to isolate and detect other highly pathogenic biomolecules with similar structural characteristics by altering the corresponding aptamer in the Fe_3_C@C conjugates.
